# Manganese in Diagnostics: A Preformulatory Study

**DOI:** 10.3390/pharmaceutics14010108

**Published:** 2022-01-03

**Authors:** Maddalena Sguizzato, Walter Pula, Anna Bordin, Antonella Pagnoni, Markus Drechsler, Lorenza Marvelli, Rita Cortesi

**Affiliations:** 1 Department of Chemical, Pharmaceutical and Agricultural Sciences (DoCPAS), University of Ferrara, I-44121 Ferrara, Italy; sgzmdl@unife.it (M.S.); walter.pula@edu.unife.it (W.P.); anna.bordin@edu.unife.it (A.B.); lorenza.mavelli@unife.it (L.M.); 2 Biotechnology Interuniversity Consortium (C.I.B.), Ferrara Section, University of Ferrara, I-44121 Ferrara, Italy; 3 Department of Environmental Sciences and Prevention, University of Ferrara, I-44121 Ferrara, Italy; antonella.pagnoni@unife.it; 4 Bavarian Polymer Institute (BPI) Keylab “Electron and Optical Microscopy”, University of Bayreuth, D-95440 Bayreuth, Germany; markus.drechsler@uni-bayreuth.de

**Keywords:** manganese, anionic liposomes, PET/MRI, lipid-based nanosystem

## Abstract

This investigation aims to find lipid-based nanosystems to be used as tools to deliver manganese for diagnostic purposes in multimodal imaging techniques. In particular, the study describes the production and characterization of aqueous dispersions of anionic liposomes as delivery systems for two model manganese-based compounds, namely manganese chloride and manganese acetylacetonate. Negatively charged liposomes were obtained using four different anionic surfactants, namely sodium docusate (SD), N-lauroylsarcosine (NLS), Protelan AG8 (PAG) and sodium lauroyl lactylate (SLL). Liposomes were produced by the direct hydration method followed by extrusion and characterized in terms of size, polydispersity, surface charge and stability over time. After extrusion, liposomes are homogeneous and monodispersed with an average diameter not exceeding 200 nm and a negative surface charge as confirmed by ζ potential measurement. Moreover, as indicated by atomic absorption spectroscopy analyses, the loading of manganese-based compounds was almost quantitative. Liposomes containing NLS or SLL were the most stable over time and the presence of manganese-based compounds did not affect their size distribution. Liposomes containing PAG and SD were instable and therefore discarded. The in vitro cytotoxicity of the selected anionic liposomes was evaluated by MTT assay on human keratinocyte. The obtained results highlighted that the toxicity of the formulations is dose dependent.

## 1. Introduction

Recently, diagnostic imaging research has led to the development of multimodal imaging techniques such as positron emission tomography (PET)/magnetic resonance imaging (MRI), which allows for metabolic information provided by PET and morphological information provided by MRI to be obtained at the same time [1,2,3].

However, the optimization of this technique has highlighted the need to design and develop specific contrast agents capable of carrying out this double and simultaneous detection [4]. A fundamental prerequisite for achieving integration between the two mentioned imaging modalities is the use of a chemically identical radioactive and paramagnetic contrast agent. In this regard, manganese (Mn) appears to be the ideal candidate as a potential bimodal contrast agent [5].

Manganese is an important transition metal belonging to the VIIB group of the periodic table, characterized by atomic number 25 and electronic configuration [Ar]3d54s2. Its oxidation state ranges from −3 to +7, but the most common forms found in living tissues are Mn^2+^ and Mn^3+^ [6], which, being endowed with paramagnetic properties, allow their possible use as contrast agents in MRI. In particular, in its bivalent state, the metal carries five unpaired electrons, producing an efficient positive contrast improvement, comparable to that obtained with Gadolinium [7]. For instance, Mn^2+^ ion is mainly employed in manganese-enhanced magnetic resonance imaging (MEMRI) used in preclinical in vivo studies [8,9]. Indeed, Mn^2+^ ion, being an analogue of the calcium ion, is able to enter within excitable cells, such as cardiac and neuronal cells, and in mitochondria of brain and liver through the voltage-dependent Ca^2+^ channels [10]. MEMRI is, therefore, an advantageous diagnostic method to visualize the activity and anatomy of those regions of the body in which Mn^2+^ accumulates [8]. It is currently mainly used to study cell structure, neuronal connections and brain function [11].

In nature, manganese is found mainly as a stable isotope ^55^Mn. Over the years, other isotopes have been synthesized, but only a few possess a half-life (>1 min) sufficient to be employed in the medical field. For instance, the isotopes ^51^Mn, ^52m^Mn and ^52g^Mn exhibit optimal properties, but the one of greatest interest for PET analysis is typified by ^52g^Mn, being characterized by a longer t_1/2_ (5–6 days) and subsequent simpler chemical manipulation as a radionuclide. Moreover, its half-life also allows the investigation of slower biological processes. Therefore, the combination of these factors gives rise to a better resolution of PET images [5].

Manganese is an essential nutrient for the human body as it is involved in a huge variety of metabolic functions, such as the regular activity of nervous, immunological and reproductive systems, the normal development of the body and as cofactor for a variety of enzymes involved in the cell protection from free radical damages (i.e., antioxidants) and in the synthesis of neurotransmitters [12].

The main source of Mn is the diet. Indeed, the U.S. Food and Drug Administration (FDA) recommends a daily dose of about 2 mg/day for adults and 1.2–1.5 mg/day for children [13]. However, even if adequate levels of Mn are essential for life, excessive exposure poses a threat to health. In fact, it has been shown that Mn at high doses is involved in the increase in cellular oxidative stress due to the variation in its oxidation state from Mn^2+^ to Mn^3+^, therefore inducing the formation of free radicals as a pro-oxidant [14]. Notably, a typical irreversible pathological condition caused by the accumulation of Mn in dopaminergic neuron-rich areas of the brain is manganism. The early stages of manganism are characterized by psychotic symptoms including compulsive and aggressive behavior, irritability, hallucinations, mood changes and intellectual deficits. In addition, extrapyramidal symptoms similar to those of Parkinson’s disease subsequently appear [6].

Currently, the neurotoxic effects are the main limiting factor for the use of Mn as a contrast agent in clinical practice. Indeed, MR relaxation times are directly proportional to the tissues concentration of Mn^2+^ ions, and the greater the Mn^2+^ amount the stronger and detectable the contrast and the better the delineation of anatomical structures [8,11]. Therefore, in order to minimize side effects, the critical point for the use of Mn as a contrast agent is the need to employ the lowest dose sufficient to be detected when using MRI.

To limit Mn toxicity, it is possible to mask the ion before its administration, using for instance Mn chelates such as mangafodipir (Teslascan^TM^) [15]. Mangafodipir is the only Mn-based contrast agent approved by the FDA for the detection of liver lesions [7]. However, in 2012, it was withdrawn from European trade for commercial and toxicity reasons [16].

Nowadays, considering the advancement of nanotechnology in diagnostic application, the encapsulation of Mn in drug delivery systems could be a promising approach to decrease its toxicity considering, at the same time, the suitability of its magnetic properties to achieve a targeted action. The use of liposomes in MRI has been explored by exploiting the binding potential of manganese able to produce complexes with inorganic species, such as arsenite [17,18], or to deliver active drugs, such as doxorubicin [19,20], after exposure to external stimuli, in particular, photothermal activation. Additionally, the use of manganese-loaded liposomes in PET is still under investigation and in the literature only a few studies are reported [21,22,23,24].

However, to our knowledge, the involvement of liposomes in the MEMRI technique has not yet been developed and their employment in the multimodal PET/MRI practice using Mn ions needs to be optimized [25,26].

**Figure 1:**
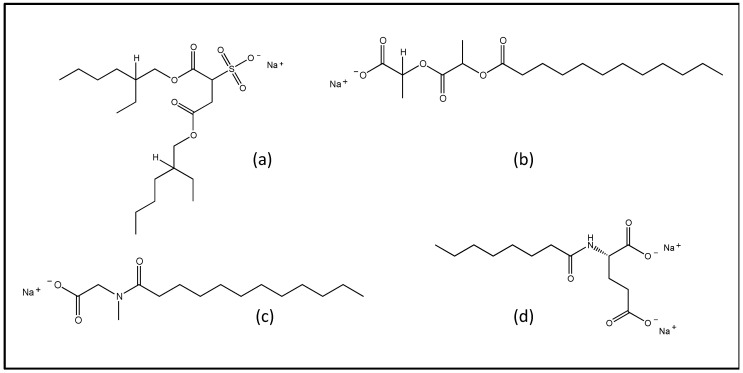
Chemical structure of anionic surfactants used in liposome composition: Sodium docusate (SD) (**a**); Protelan AG8 (PAG) (**b**); N-lauroylsarcosine sodium salt (NLS) (**c**); sodium lauroyl lactylate (SLL) (**d**).

Taking these data in mind, a potential strategy to take advantage of manganese’s bimodal contrast properties but limiting its side effects, could be represented by the carrying of Mn ions into liposomes, known for their wide range of applications, including diagnostics and imaging, and targeted delivery [27]. In particular, anionic liposomes have been investigated with the aim of exploiting the possibility of ionic interaction between the manganese and the vesicular surface to try to increase the loading of the manganese itself in the formulation. Furthermore, due to their extravasation, anionic liposomes can be useful in the diagnostic field as probe transporters in the extravascular compartment of a tumor [28]. Accordingly, the present study focuses on a preformulatory study of anionic liposomes as delivery systems for manganese. In particular, two model compounds of manganese with different water solubility, such as manganese chloride (MC) and manganese acetylacetonate (MA), have been considered. Negatively charged liposomes were produced using the direct hydration method followed by extrusion [29], in order to electrostatically bind manganese positive ions thanks to the negative charge granted by the surfactant. In particular, four different anionic surfactants were considered, namely sodium docusate (SD), N-lauroylsarcosine sodium salt (NLS), Protelan AG8 (PAG) and sodium lauroyl lactylate (SLL) (Figure 1).

The obtained formulations were then characterized in terms of size, surface charge, efficiency of encapsulation and stability over time. In addition, the anionic liposomal systems with optimal characteristics were subjected to an in vitro antiproliferative test on human cells by means of an MTT assay.

## 2. Materials and Methods

### 2.1. Materials

Manganese chloride tetrahydrate (MC), manganese acetylacetonate (MA), sodium docusate (SD) and cholesterol (CH) were from Merck-Aldrich (Milano, Italy). N-Lauroylsarcosine sodium salt (NLS) was from Fluka Chemie AG. Protelan AG8 (PAG) and sodium lauroyl lactylate (SLL) were from Zschimmer & Schwarz Italiana S.p.A. (Vercelli, Italy) and Stepan Europe S.A.S. (Voreppe, France), respectively. Finally, soybean phosphatidylcholine Phospholipon 90G (PC) was from Lipoid AG (Steinhausen, Switzerland). Solvents of analytical grade were from Merck Serono S.p.A. (Rome, Italy). All other materials and solvents of the high purity grade were from Sigma-Aldrich (St Louis, MO, USA).

### 2.2. Liposomes Preparation

Anionic liposomes were prepared by direct hydration followed by extrusion. An organic solution of CH_2_Cl_2_ and methanol (1:1 *v/v*) containing PC, CH and the anionic surfactant (AS) in a 4:2:1 molar ratio was prepared. Afterwards the organic mixture was evaporated under vacuum (70 bar, rotation speed 3 for 40–45 min) by mean of a Rotavapor R-200 (Buchi Italia, Cornaredo, Italy) and the obtained film was hydrated with water, vortexed and sonicated for 5 min. The addition of manganese-based compounds (500 μM) was carried out in the selected formulation either in organic phase (MA, lipophilic) or in water (MC, hydrophilic) during the hydration step. Table 1 reports the composition of the prepared liposomal formulations.

Liposomes were then subjected to extrusion leading to vesicles with a homogeneous size distribution [30]. Precisely, each liposome dispersion was extruded five-fold through two stacked polycarbonate filters with 0.2 µm pore size (Nucleopore Corp, Pleasanton, CA, USA) supported by polyester drain disk using an Extruder (Lipex Biomembranes, Vancouver, BC, Canada) and 10–20 bars nitrogen pressure [31]. After the process, liposomes were collected and stored for further studies.

### 2.3. Cryo-Transmission Electron Microscopy (Cryo-TEM)

Liposome samples were vitrified and transferred to a Zeiss EM922Omega transmission electron microscope for imaging using a cryoholder (CT3500, Gatan Inc., Pleasanton, CA, USA), as previously described [32,33]. Sample temperature was maintained below −175 °C throughout the visualization. Specimens were examined with doses of about 1000–2000 e/nm^2^ at 200 kV. Images were recorded digitally by CCD camera (UltraScan 1000, Gatan Inc., Pleasanton, CA, USA) using GMS 1.4 software (Gatan Inc., Pleasanton, CA, USA) as image processing system.

### 2.4. Photon Correlation Spectroscopy (PCS)

Liposome size was measured by mean of Zetasizer Nano S90 (Malvern Instr., Malvern, UK) equipped with a 5 mW helium neon laser with a wavelength output of 633 nm on aqueous diluted liposome samples (1:20 by volume). Plasticware was cleaned with detergent washing and rinsed twice with milliQ water. Measurements were made at 25 °C at an angle of 90°, run time around 180 s. Data were interpreted by the “CONTIN” method [34].

### 2.5. ζ Potential Measurements

To determine the surface charge of the liposomes, the measurement of the zeta potential (ζ) was carried out. For this purpose, the device Zetasizer Ultra (Malvern Panalytical Ltd., Malvern, UK) was used. All the samples were diluted in disposable capillary cells (DTS 1080, Malvern) with deionized water (1:20 *v/v*) and were subjected to analysis, conducted at 25 °C. Values were obtained from three independent experiments performed in triplicate.

### 2.6. Atomic Absorption Spectrophotometry (AAS)

AAS is an analytical technique used for quantitative and qualitative determination of metal ions in solution. Therefore, each sample was subjected to the AAS to determine the concentration of Manganese actually present. The encapsulation efficiency (EE) of manganese in LP-NLS and LP-SLL was determined by ultracentrifugation; specifically, 500 μL samples were loaded in a centrifugal filter (Microcon centrifugal filter unit YM-10 membrane, NMWCO 10 kDa, Sigma-Aldrich, St. Louis, MO, USA) and subjected to ultracentrifugation (Spectrafuge™ 24D Digital Microcentrifuge, Woodbridge, NJ, USA) at 8000 rpm for 20 min. Then, the lipid phase, the aqueous phase and an aliquot from a formulation not subjected to ultracentrifugation were analyzed. All measurements were made with an AAS device (Analyst 800, Perkin-Elmer, Shelton, CT, USA) equipped with a Zeeman background correction system and an electrothermal atomizer with a transversely heated graphite tube. The measure was performed at a wavelength equal to 279.5 nm. Each sample was prepared twice and subjected to analysis, the analyzed volume being 20 µL. For each condition, the average of the absorbance values obtained was calculated and the manganese concentration was obtained by comparison with a calibration curve obtained after measuring known concentrations of the metal ion [35]. The EE was determined as follows

EE = [Mn_LIPID_ (mM)/Mn_LIPID_ (mM) + Mn_AQUEOUS_ (mM)] × 100 (1)

where Mn_LIPID_ corresponds to the concentration of manganese in the lipid phase measured by AAS, and Mn_AQUEOUS_ is the concentration of manganese in the aqueous phase measured by AAS.

### 2.7. Interaction between Manganese-Based Compounds and Liposomes

FT-IR spectroscopy was employed to study the interaction of selected liposomes with manganese-based compounds. Precisely, plain-LP and LP-SLL empty or loaded with increasing concentrations of MC or MA were considered. Analyses were performed using a Bruker Vertex 70 spectrophotometer (Bruker Italia srl, Milano, Italy) using the diffuse reflectance technique on a solid KBr sample. Samples were prepared depositing 10 μL of the liposomal dispersions on 50–100 mg of anhydrous KBr and a tablet was compressed for analysis, after obtaining a homogeneous mixture [36].

### 2.8. Cell Viability Test

Cell viability was evaluated using MTT test [37] on HaCaT cells grown in Dulbecco’s modified Eagle’s medium High Glucose (DMEM) (Lonza, Milan, Italy), supplemented with 10% FBS (fetal bovine serum), 100 U/mL penicillin, 100 μg/mL streptomycin and 2 mM L-glutamine. Cells were incubated at 37 °C for 24 h in 95% air/5% CO_2_ until 80% confluence. Liposome formulations were dispersed in cell culture medium and diluted 1:50, 1:100, 1:200, 1:500, corresponding to liposome concentrations of 0.5, 0.25, 0.125, 0.05 mg/mL, respectively. Plain-LP were added following the same dilution step used for manganese-loaded LP in order to reach the same content in terms of lipid nanoparticles within the wells. Seeded cells were exposed to the selected formulations for 24 h, and, after complete removal of the treatment, 110 μL of MTT (0.5 mg/mL) was added and incubated for 4 h. The conversion of MTT solution into a violet colored formazane was obtained after the addition, incubation (15 min) and shaking of 100 μL of DMSO. The solution absorbance, proportional to the number of living cells, was measured using a spectrophotometer at 590 nm and converted into percentage of viability.

Statistical analysis was performed by the analysis of variance (ANOVA). The level of significance was taken at *p*-values < 0.05.

## 3. Results and Discussion

### 3.1. Liposome Production and Characterization

In this study, the attention was focused on the development of anionic liposomal systems able to carry the manganese ion (i.e., Mn^2+^ or Mn^3+^), characterized by high toxicity, with the aim to avoid its release into biological fluids before reaching the site of interest [38,39].

**Figure 2:**
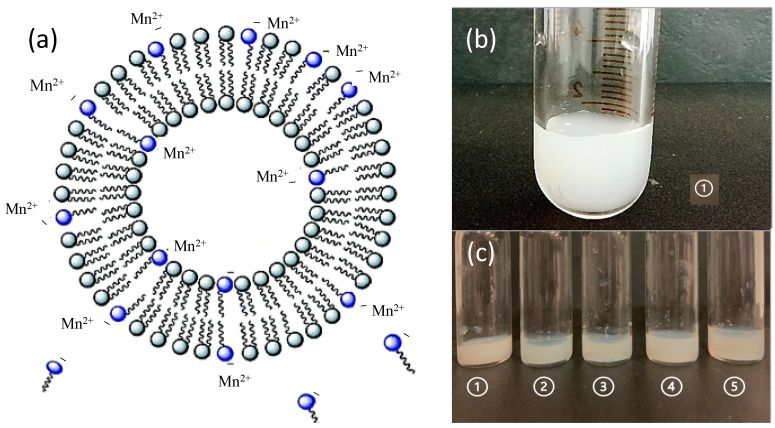
Schematic representation of the arrangement of surfactants in the phospholipid bilayer and of their possible electrostatic interaction with the manganese ion, namely Mn^2+^ or Mn^3+^ (**a**) and macroscopic aspect of the produced liposomal suspensions before (**b**) and after extrusion (**c**), 1: plain-LP; 2: LP-SD; 3: LP-NLS; 4: LP-PAG; 5: LP-SLL.

In this regard, a preformulative study was conducted. In particular, in order to give the vesicle a negative surface charge and take advantage of the electrostatic interaction possibly established between the polar head of the negatively charged surfactant and manganese ion, the addition of different AS to the lipid composition of the liposomes was investigated. Figure 2 highlights the arrangement of the surfactants within the phospholipid bilayer of the liposomes: the hydrophilic heads are arranged in such a way as to be in contact with the aqueous medium, while the lipophilic tails remain in close proximity to each other. The electrostatic interaction between the polar portion with negative charge and the positively charged Mn^2+^ or Mn^3+^, can occur both on the outer surface of the liposomal vesicles and inside the vesicles.

Negatively charged liposomes were produced using the direct hydration method followed by extrusion [29]. As anionic surfactants, SD, NLS, PAG and SLL (Figure 1) were used.

From the macroscopic point of view, the aspect of liposomal dispersions before extrusion exhibit a milky color (Figure 2b), which becomes more transparent in the extruded formulations (Figure 2c). A further increase in the transparency of the extruded liposomal dispersions may also be due to the presence of the anionic surfactants tested (Figure 2c).

**Figure 3:**
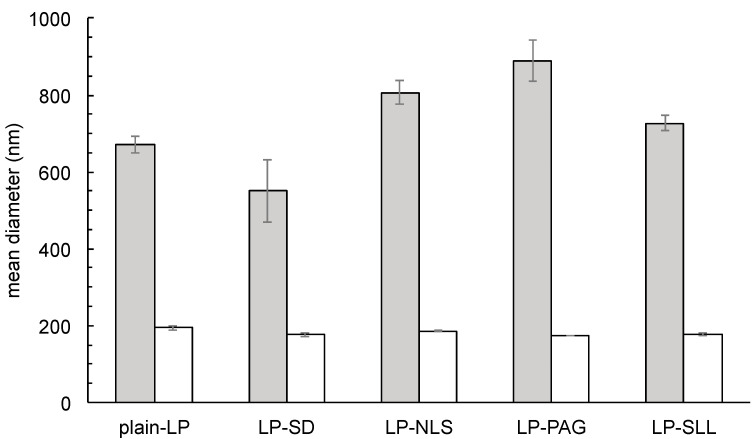
Mean diameter of the produced anionic liposomes before (grey) and after (white) extrusion through 200 nm pore size polycarbonate filters as determined by PCS, *p*-values < 0.05.

The unloaded liposomal dispersions were analyzed in term of size and charge by evaluating their average diameter, polydispersion index and ζ potential. The measurements were carried out before and after the extrusion process through polycarbonate filters with 200 nm pore size, and the obtained results are summarized in Table 2 and Figure 3. As expected, from the analysis of the data, it is evident that, after extrusion, the average diameter of liposomes decreases, reflecting the pore size of the polycarbonate filter used for the extrusion. In addition, the presence of AS within the lipid bilayer has an effect, albeit not marked, on the size of the anionic vesicles, which are slightly smaller than plain-LP (Table 2). Moreover, the measure of ζ potential confirmed the presence of a net negative surface charge for those liposomal dispersions carrying AS (Table 2), evidencing that the extrusion process slightly affected the net charge of anionic liposomes in agreement with the literature data [40]. Furthermore, each sample displays a ζ potential greater than 30 mV in absolute value, thus, the dispersion is particularly stable in view of the electrostatic repulsion developed between the charges of the same sign avoiding the agglomeration of the liposomal vesicles [41]. The comparison between the values of the ζ potential (Table 2), highlights the different influence of the AS addition on the liposomes. In particular, the significant increase in ζ potential after the addition of SD, NLS or SLL led to a greater charge repulsion and, consequently, an improved stability of the system [42]. On the other hand, PAG, unlike other surfactants, did not impart a greater surface charge as compared to plain-LP and, consequently, the stability of the liposomal dispersion was limited, as confirmed by the formation of a large aggregate in a short time (7 days) (see Figure S1).

Concerning the polydispersity index, the extruded preparations show much lower values than those of the corresponding dispersion before extrusion, indicating once again the effect of the extrusion process on obtaining a dimensional homogeneity of dispersed vesicles (Table 2 and Figure 3).

**Figure 4:**
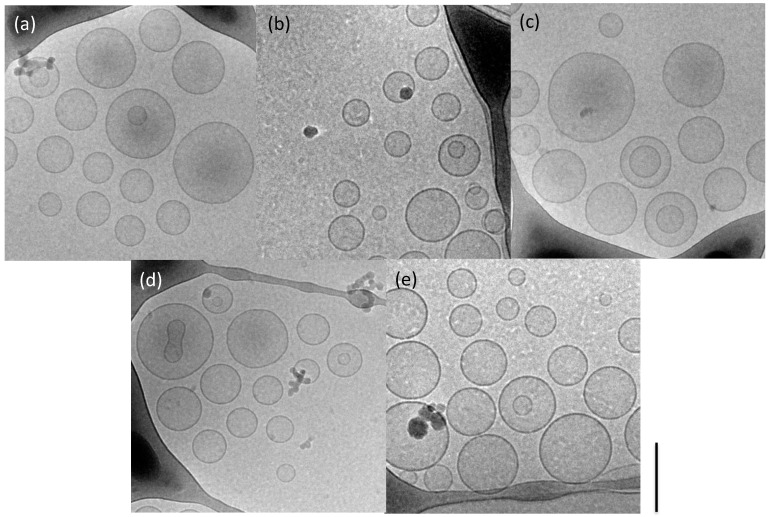
Cryo-TEM images of the produced unloaded anionic liposomes visualized after extrusion. Plain-LP (**a**), LP-SD (**b**), LP-NLS (**c**), LP-PAG (**d**) and LP-SLL (**e**), Bar corresponds to 250 nm.

Furthermore, the morphology of liposome dispersions was investigated by cryo-TEM, and the obtained images reported in Figure 4 confirmed that the extrusion process allowed the obtaining of quite homogeneous dispersions, in agreement with PCS analysis, mainly characterized by unilamellar liposomes [43]. Hence, the morphological analysis corroborated the monomodal distribution of vesicles evidenced by PdI data lower than 0.3 [44].

### 3.2. Selection of Liposome Compositions for Mn Loading

The choice of the most suitable composition for the subsequent manganese loading was made on the basis of a dimensional stability study lasting one month on the four extruded anionic liposomal preparations.

From the analysis of data reported in Table 3, it is evident that liposomes prepared with NLS and SLL were the most stable over time. Indeed, the results obtained show that both the average diameter of the liposomes and their PdI values remained fairly unchanged in all anionic liposome types over a period of thirty days, with the exception of formulations with Protelan-AG8. In this last case, the measure of the size was stopped after seven days due to the formation of a large white aggregate. As regards the other three formulations, the comparison of the dimensional trends shows that in the first two weeks the dimensions of the liposomes oscillated in a range between 160 and 185 nm. Subsequently, over time it was observed that the dimensions of LP-SD were considerably reduced compared to the other preparations, also showing a high increase in the polydispersity index on the thirtieth day. In addition, from a macroscopic point of view, the appearance of the preparations, after 30 days, shows that LP-NLS and LP-SLL remained stable, while LP-SD and LP-PAG showed aggregates of white and yellow color, respectively (Figure S1). Taken together, these results demonstrate that liposomes containing PAG and SD showed high instability, and therefore were excluded from the subsequent experiments of manganese loading.

### 3.3. Production and Characterization of Manganese-Loaded Liposomes

Mn-loaded LP-SLL and LP-NLS were produced as described in the Materials and Methods section. In particular, two different model manganese-based compounds were used, namely MC and MA, in reason of their different solubility in water.

**Figure 5:**
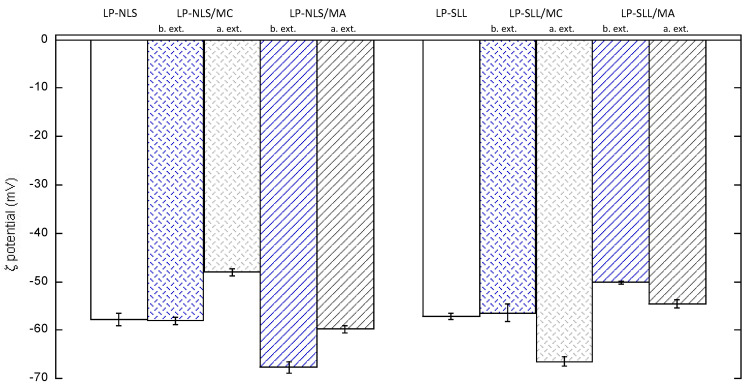
ζ potential values of unloaded or manganese-loaded NLS and SLL liposomes.

Figure 5 and Table 4 summarize the effect of the presence of MC and MA on the charge and size of the LP-NLS and LP-SLL formulations, before and after the extrusion process.

It was found that after loaded liposomes’ production, the presence of MC and MA did not induce significant changes in ζ potential values, which remained between −57 mV and −67 mV. However, a slight reduction in ζ potential was observed in manganese-loaded liposomal systems that had instead undergone the extrusion process. Nonetheless, the charge reduction can be considered acceptable in comparison with unloaded liposomes, being higher than 45 mV in absolute value.

**Figure 6:**
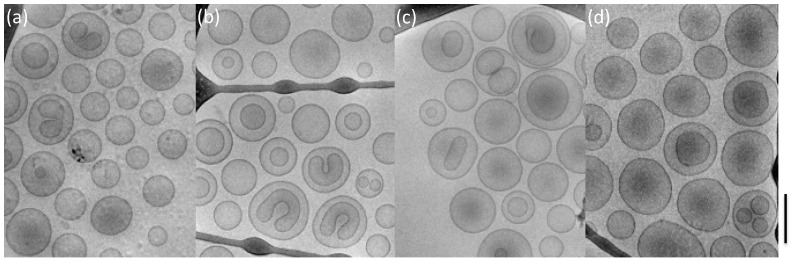
Cryo-TEM images of the produced manganese-loaded NLS and SLL liposomes. LP-NLS-MC (**a**), LP-NLS-MA (**b**), LP-SLL-MC (**c**) and LP-SLL-MA (**d**). Bar corresponds to 200 nm.

Concerning the size, the reductive effect of the extrusion process on distribution was confirmed, leading to the achievement of homogeneous dispersions where the presence of manganese did not affect the size distribution. This result corroborated the cryo-TEM investigation (Figure 6). Indeed, the cryo-TEM visualization showed that all formulations have vesicular appearance consisting of a double-layer structure typical of liposomal systems.

In addition, independently from the type of anionic surfactant used, a mixture of bilamellar and unilamellar vesicles were detected. Moreover, as compared to the corresponding unloaded formulation (Figure 4), the presence of manganese did not induce differences in the morphology of vesicles, allowing them to obtain stable formulations.

#### 3.3.1. Mn Encapsulation Efficiency

**Figure 7:**
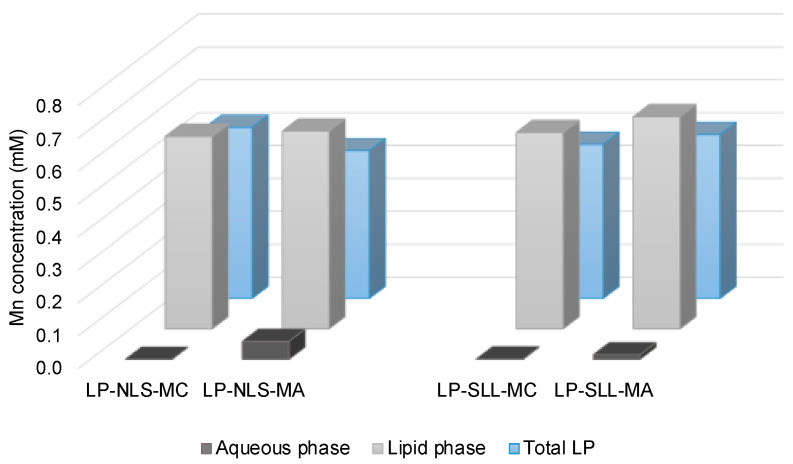
MC and MA concentration in the aqueous phase (dark grey), in the lipid phase (light grey) of LP-NLS and LP-SLL after ultracentrifugation, and in the formulations before ultracentrifugation (light blue). Data are the means of three independent experiments conducted in duplicate.

The encapsulation efficiency of MC and MA in LP-NLS and LP-SLL was evaluated after the extrusion process by AAS analysis. In particular, in order to evaluate manganese loading based on the electrostatic binding with the anionic surfactant arranged in the phospholipid bilayer, loaded liposomes were subjected to ultracentrifugation, obtaining the separation of the lipid and the aqueous portions [45]. Figure 7 shows the manganese concentration obtained in the total formulation after extrusion, and in the lipid and aqueous phases of centrifuged liposomes.

The results show that both MC and MA were retained by the lipid portion of LP-NLS and LP-SLL, while in the aqueous phase, manganese concentration was almost zero. Comparing these data with the concentration in the whole formulation, it should be underlined that the manganese encapsulation was almost quantitative. In particular, in the case of MC, the EE in LP-NLS and LP-SLL were, respectively, 99.83% and 99.72%, while in the case of MA they were 91.66% and 97.88%, respectively, calculated as reported in Section 2.5 (Equation (1)). The slight decrease in MA loading can be ascribed to the lipophilic nature of the molecule.

Therefore, these results confirmed the ability of liposomes to carry manganese ions, corroborating their potential electrostatic binding with the negative charges of surfactants in the lipid bilayer.

#### 3.3.2. Interaction between Manganese-Based Compounds and Liposomes

To study the interaction of liposomes with manganese-based compounds, i.e., MC and MA, FT-IR spectroscopy analyses were carried out on selected formulations on the basis of AAS results. Precisely, plain-LP and anionic LP-SLL, empty or loaded with increasing concentrations of MC or MA, were considered.

**Figure 8:**
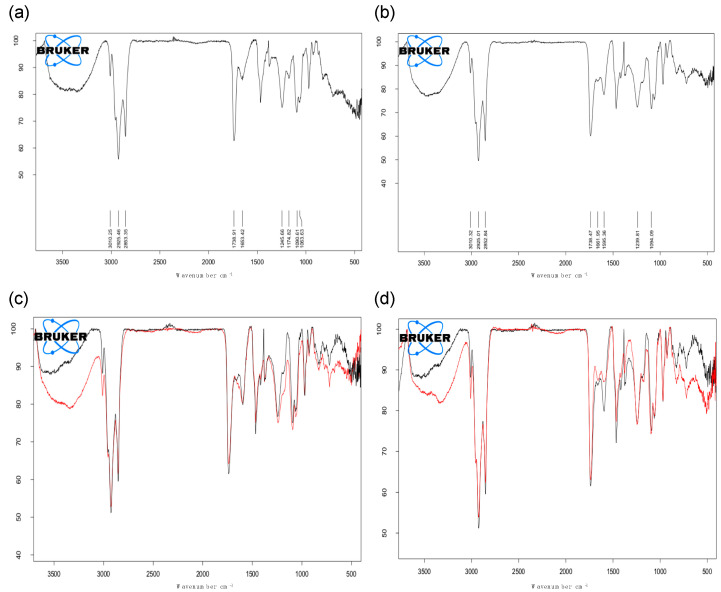
FT-IR spectra of plain-LP (**a**), LP-SLL (**b**), LP-SLL (black) overlapped to LP-SLL-MC (red) (**c**) and LP-SLL (black) overlapped to LP-SLL-MA (red) (**d**).

Firstly, the analysis of plain-LP and LP-SLL highlighted the main functional peaks ascribable to the liposomes structure, that is phosphatidylcholine (PC), cholesterol (CH) or to the anionic surfactant. As shown in Figure 8a (plain-LP IR spectrum) and 8b (LP-SLL IR spectrum), the two spectra are almost superimposable and most of the signals are referred to functional groups of PC and CH that characterize the vesicles. In particular, in the region 3200–3600 cm^−1^, the O-H is representative of the phosphate group of PC, the hydroxyl group of CH and the carboxylic group of SLL, confirming the inclusion of SLL in the lipid bilayer [46]. Additionally, in the LP-SLL spectrum a characteristic peak (1595 cm^−1^) refers to the carboxylate ion −COO^−^ of the anionic surfactant.

Furthermore, the preliminary characterization of the unloaded liposomes allowed information to be gained about their interaction with manganese compounds by comparing the different spectra. Indeed, the electrostatic or covalent binding between the vesicles and MC or MA results in the variation in absorption frequency. In particular, increasing the amount of MC and MA, namely 0.5, 1, 2, 4, 8 and 16 mM, was considered with the aim of studying the influence of the concentration on the type of liposome interaction, in order to select the final concentration detectable by the FT-IR analysis. Indeed, considering the surfactant amount was much higher than those of MC and MA in the formulation, the signal of the unloaded liposomes covers the manganese signal, avoiding the evaluation of the interaction. The overlapped spectra of LP-SLL and LP-SLL-MC or LP-SLL-MA are displayed in Figure 8c,d respectively, with the highest manganese concentration tested (16 mM).

With regard to LP-SLL-MC, the higher the concentration of MC, the higher the intensity of the peak between 1600–1640 cm^−1^. As mentioned above, the peak of the −COO^−^ group of SLL appeared in this region, hence, the variation in the absorption frequency evidences the electrostatic nature of the interaction between MC and the polar head of the surfactant located on the surface of the phospholipid bilayer [47]. Accordingly, this result is in agreement with the hydrophilic nature of MC, arranged in the internal region of liposomes.

In the case of LP-SLL-MA, the spectrum was completely superimposable with that of LP-SLL, and, in particular, in the region 1600–1640 cm^−1^ referring to SLL and MA, no variations in the signals were detected. This evidence indicates that the electrostatic binding with the polar head of the surfactant did not occur. Indeed, MA, being lipophilic, did not interact with the surface, although it was entrapped in the phospholipid bilayer, and any variations in the peaks were covered by the PC and CH signals much higher than manganese concentration [48].

The FT-IR analysis allowed corroboration of the high EE of MC and MA in anionic liposomes, describing the type of binding that occurred in the case of hydrophilic or lipophilic compounds.

### 3.4. In Vitro Effect of Produced Liposomal Formulations on Cultured Cells

**Figure 9:**
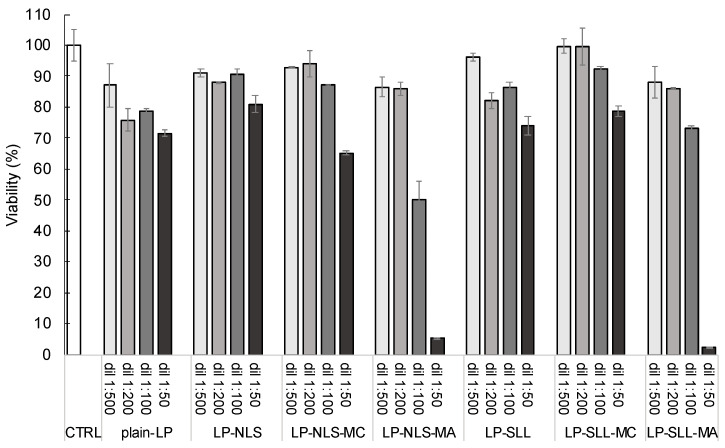
In vitro effect on HaCaT cell proliferation, performed by MTT test, of plain-LP, anionic LP-NLS and LP-SLL unloaded and loaded with MC and MA. Data are the mean of three independent experiments ± s.d. conducted in triplicate, obtained *p*-values always <0.01 vs. CTRL.

In order to evaluate the in vitro activity on cell proliferation of the selected formulations, the MTT test was performed on HaCaT cells, as a model human non-tumor cell line. Plain-LP, and unloaded and manganese-loaded LP-NLS and LP-SLL were tested at different concentrations after appropriate dilutions, and the effect was compared to those of untreated cells used as control. The results were expressed as the percentage of cell viability with respect to control cells (100% viable) and are summarized in Figure 9.

The obtained results demonstrate that all of the formulations are characterized by a dose-dependent antiproliferative effect on HaCaT cells cultured in vitro. Hence, the higher the concentration of formulation, the lower the viability. In particular, plain-LP and both unloaded anionic formulations did not affect cell proliferation for all concentrations tested. Moreover, it should be underlined that cell viability was not only influenced by the LP concentration, but also by the presence of the anionic surfactant. The viability of cells treated with charged liposomes was slightly higher than those treated with plain-LP. This effect could be ascribed to the possible electrostatic repulsion between the negative charge and the membrane macromolecules, leading to a reduced cell–liposome interaction with a subsequent reduction in the LP antiproliferative effect [49,50].

Furthermore, LP-NLS or LP-SLL loaded with two manganese compounds and characterized by different levels of solubility in water were considered. The actual concentration of manganese in each formulation was determined by the mean of atomic absorption spectroscopy, demonstrating that the manganese compounds were retained almost completely within liposomes. As reported in Figure 9, MC-loaded LP did not affect cell viability compared to unloaded formulations. On the other hand, MA-loaded LP showed a higher antiproliferative effect with respect to MC-loaded LP. It is probable that the lipophilic nature of MA contributes to an increase in the lipophilicity of the entire system affecting the interaction with cells. Indeed, at higher concentrations, the viability undergoes a tremendous decrease, reaching 10%.

Taking into consideration the results, further experiments need to be performed in order to elucidate the optimal concentration of manganese in terms of loading capacity and bioavailability, possibly using other non-tumor human cell lines, such as L929 fibroblast.

## 4. Conclusions

The present study is still currently in progress; however it is possible to propose some indications for future researches aimed at investigating the possible use of manganese for diagnostic applications.

This work underlined the importance of technological screening in the design of anionic liposomes carrying manganese using a simple preparation method. In particular, morphological and dimensional analyses, together with the loading capacity and in vitro activity, gave the chance to select the composition to be used for future investigations. In particular, FT-IR analyses allowed for speculation as to those aspects that were related to the interaction of manganese with the negative charges of the surfactant or with the phospholipid bilayer. These peculiar behaviors need to be further studied by determining the magnetic properties of the obtained liposomal system, a relevant factor for their potential application in diagnostic imaging.

## Figures and Tables

**Table 1:** Liposome composition.

Dispersion Acronym	PC (mg/mL)	CH (mg/mL)	Anionic Surfactant (mg/mL)
**plain-LP**	20.0	4.95	-
**LP-SD**	18.0	4.00	2.50
**LP-NLS**	18.5	4.60	1.75
**LP-PAG**	18.5	4.50	2.00
**LP-SLL**	18.5	4.50	2.00

**Table 2:** Liposomes polidispersity index and ζ potential measured before and after extrusion.

Dispersion Acronym	before Extrusion		after Extrusion	
	PdI ± s.d.	ζ Potential (mV)	PdI ± s.d.	ζ Potential (mV)
**plain-LP**	0.12 ± 0.03	−36.92 ± 1.29	0.09 ± 0.02	−29.17 ± 0.83
**LP-SD**	0.53 ± 0.02	−68.21 ± 1.23	0.11 ± 0.03	−63.50 ± 1.27
**LP-NLS**	0.49 ± 0.04	−59.77 ± 1.04	0.10 ± 0.01	−57.15 ± 1.26
**LP-PAG**	0.59 ± 0.05	−40.29 ± 0.44	0.12 ± 0.02	−36.29 ± 0.42
**LP-SLL**	0.48 ± 0.04	−61.12 ± 0.52	0.09 ± 0.02	−57.10 ± 0.64

**Table 3:** Liposome mean size and polydispersity index (PdI) during one month, as determined by PCS.

Time (Day)	LP-SD		LP-NLS		LP-PAG		LP-SLL	
	Mean Size (nm ± s.d.)	PdI ± s.d.	Mean Size (nm ± s.d.)	PdI ± s.d.	Mean Size (nm ± s.d.)	PdI ± s.d.	Mean Size (nm ± s.d.)	PdI ± s.d.
0	175.81 ± 4.51	0.11 ± 0.02	185.31 ± 2.03	0.10 ± 0.01	173.62 ± 0.62	0.12 ± 0.02	176.91 ± 3.29	0.09 ± 0.02
1	176.08 ± 4.81	0.11 ± 0.01	177.83 ± 2.62	0.10 ± 0.01	173.28 ± 5.60	0.10 ± 0.01	163.77 ± 2.71	0.13 ± 0.02
3	172.02 ± 6.13	0.12 ± 0.01	177.94 ± 3.82	0.09 ± 0.00	172.79 ± 4.73	0.10 ± 0.01	159.91 ± 0.68	0.12 ± 0.01
7	174.89 ± 5.32	0.12 ± 0.02	182.02 ± 5.21	0.11 ± 0.01	174.01 ± 5.42	0.09 ± 0.01	174.02 ± 5.37	0.10 ± 0.01
10	173.57 ± 3.71	0.11 ± 0.01	177.70 ± 4.33	0.10 ± 0.01	n.d.	n.d.	160.92 ± 3.21	0.12 ± 0.01
15	173.88 ± 4.22	0.11 ± 0.02	179.31 ± 0.93	0.12 ± 0.01	n.d.	n.d.	176.49 ± 5.13	0.13 ± 0.01
30	123.29 ± 7.12	0.31 ± 0.01	178.22 ± 1.74	0.09 ± 0.01	n.d.	n.d.	171.03 ± 4.78	0.11 ± 0.01

n.d.: not determinable.

**Table 4:** Size of Mn-loaded LP-SLL and LP-NLS measured before and after extrusion.

Dispersion Acronym	before Extrusion		after Extrusion	
	Mean Size (nm ± s.d.)	PdI ± s.d.	Mean Size (nm ± s.d.)	PdI ± s.d.
**LP-NLS-MC**	796.41 ± 28.04	0.41 ± 0.03	172.91 ± 3.81	0.12 ± 0.02
**LP-NLS-MA**	703.92 ± 22.51	0.46 ± 0.02	176.82 ± 4.91	0.09 ± 0.02
**LP-SLL-MC**	693.23 ± 18.33	0.48 ± 0.01	192.51 ± 4.13	0.13 ± 0.02
**LP-SLL-MA**	757.52 ± 32.51	0.48 ± 0.01	193.33 ± 1.54	0.12 ± 0.03

## Supplementary Materials

The following supporting information can be downloaded at: https://www.mdpi.com/article/10.3390/pharmaceutics14010108/s1. Figure S1: Macroscopic appearance of LP-SD (a), LP-NLS (b), LP-PAG (c) and LP-SLL (d) 30 days after extrusion.
