# Supplementary material for: Manganese in Diagnostics: A Preformulatory Study
Source: Pharmaceutics. 2022 Jan 3;14(1):108. doi: 10.3390/pharmaceutics14010108 (PMC8780490; doi:10.3390/pharmaceutics14010108)
Supplement: Supplementary file 1 [file pharmaceutics-14-00108-s001.zip › pharmaceutics-1511320-supplementary.pdf]

pharmaceutics

Supplementary Materials: Manganese in Diagnostics: A Preformulatory Study

Maddalena Sguizzato, Walter Pula, Anna Bordin, Antonella Pagnoni, Markus Drechsler, Lorenza Marvelli and Rita Cortesi

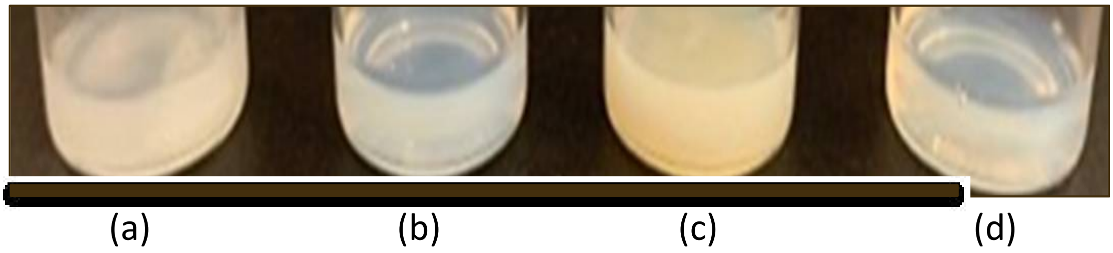

Figure S1: Macroscopic appearance of LP-SD (**a**), LP-NLS (**b**), LP-PAG (**c**) and LP-SLL (**d**) 30 days after extrusion.
